# Supplementary material for: Scoping Review of Climate Change Adaptation Interventions for Health: Implications for Policy and Practice
Source: Int J Environ Res Public Health. 2024 Nov 26;21(12):1565. doi: 10.3390/ijerph21121565 (PMC11675531; doi:10.3390/ijerph21121565)
Supplement: Supplementary file 1 [file ijerph-21-01565-s001.zip › Figure S1.pdf]

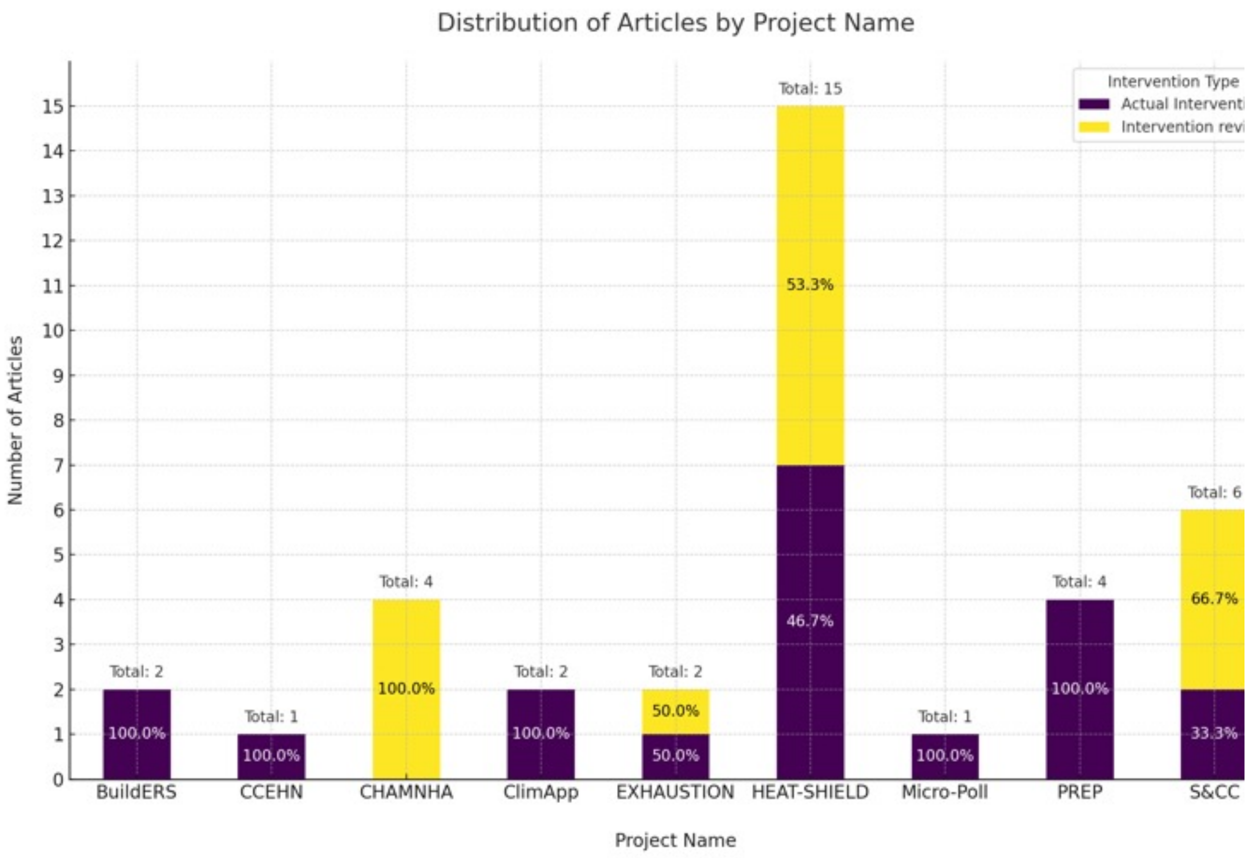
Distribution of Articles by Project Name
Intervention Type
Actual Interventi
Intervention revi
Number of Articles
Project Name
BuildERS
CCEHN
CHAMNHA
ClimApp
EXHAUSTION
HEAT-SHIELD
Micro-Poll
PREP
S&CC
Total: 2
Total: 1
Total: 4
Total: 2
Total: 2
Total: 15
Total: 1
Total: 4
Total: 6
100.0%
100.0%
100.0%
100.0%
50.0%
50.0%
53.3%
46.7%
100.0%
100.0%
66.7%
33.3%

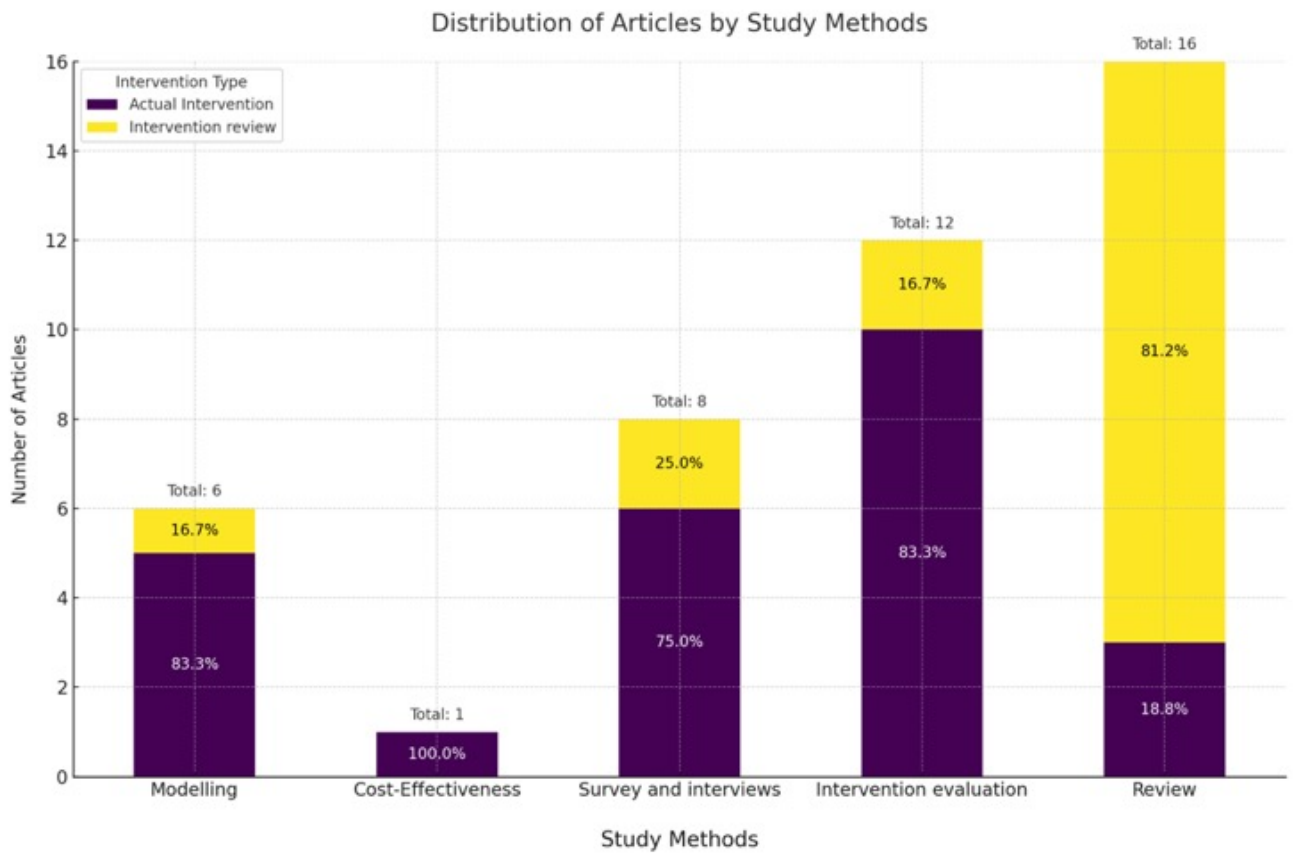
Distribution of Articles by Study Methods
Intervention Type
Actual Intervention
Intervention review
Number of Articles
Study Methods
Modelling
Cost-Effectiveness
Survey and interviews
Intervention evaluation
Review
Total: 6
Total: 1
Total: 8
Total: 12
Total: 16
16.7%
83.3%
100.0%
25.0%
75.0%
16.7%
83.3%
81.2%
18.8%

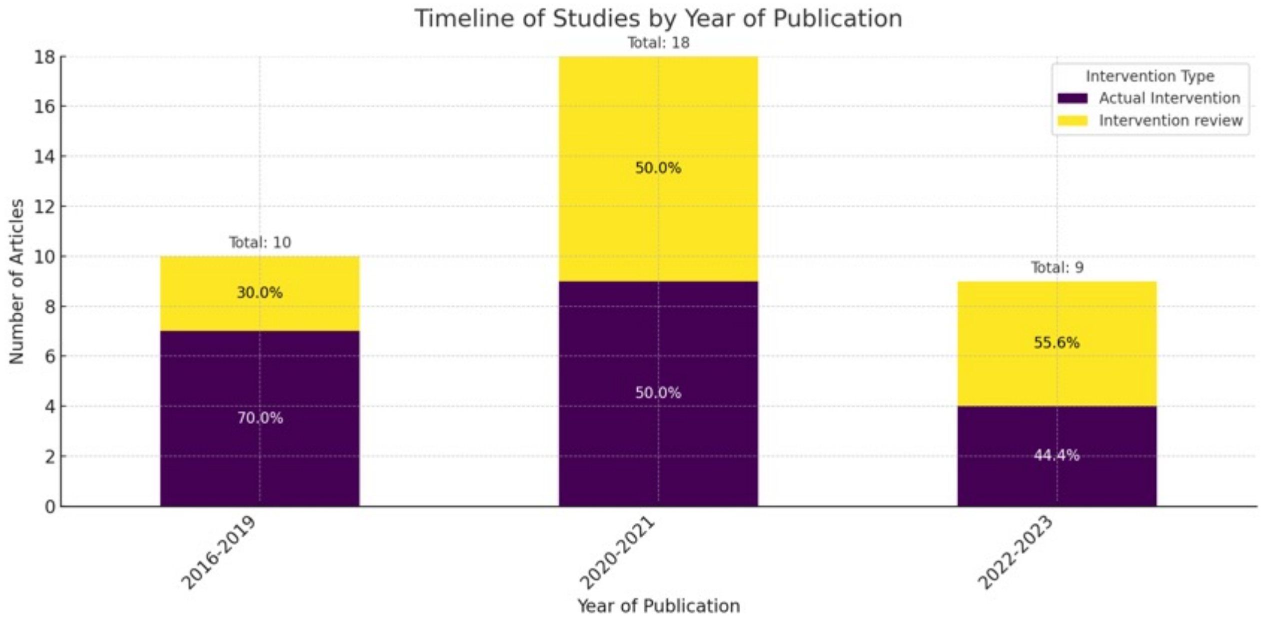
Timeline of Studies by Year of Publication
Intervention Type
Actual Intervention
Intervention review
Number of Articles
Year of Publication
2016-2019
2020-2021
2022-2023
Total: 10
Total: 18
Total: 9
30.0%
70.0%
50.0%
50.0%
55.6%
44.4%

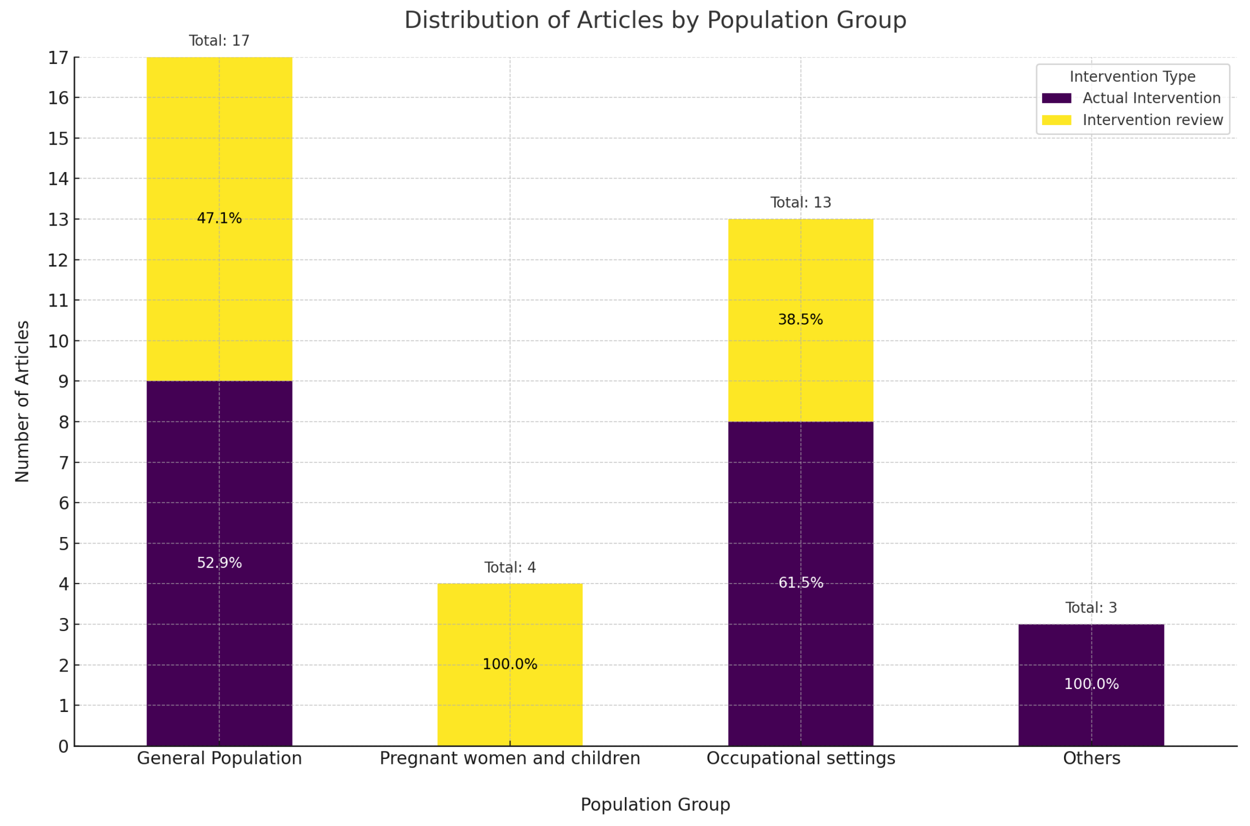
Distribution of Articles by Population Group
Intervention Type
Actual Intervention
Intervention review
Number of Articles
Population Group
General Population
Pregnant women and children
Occupational settings
Others
Total: 17
Total: 4
Total: 13
Total: 3
47.1%
52.9%
100.0%
38.5%
61.5%
100.0%

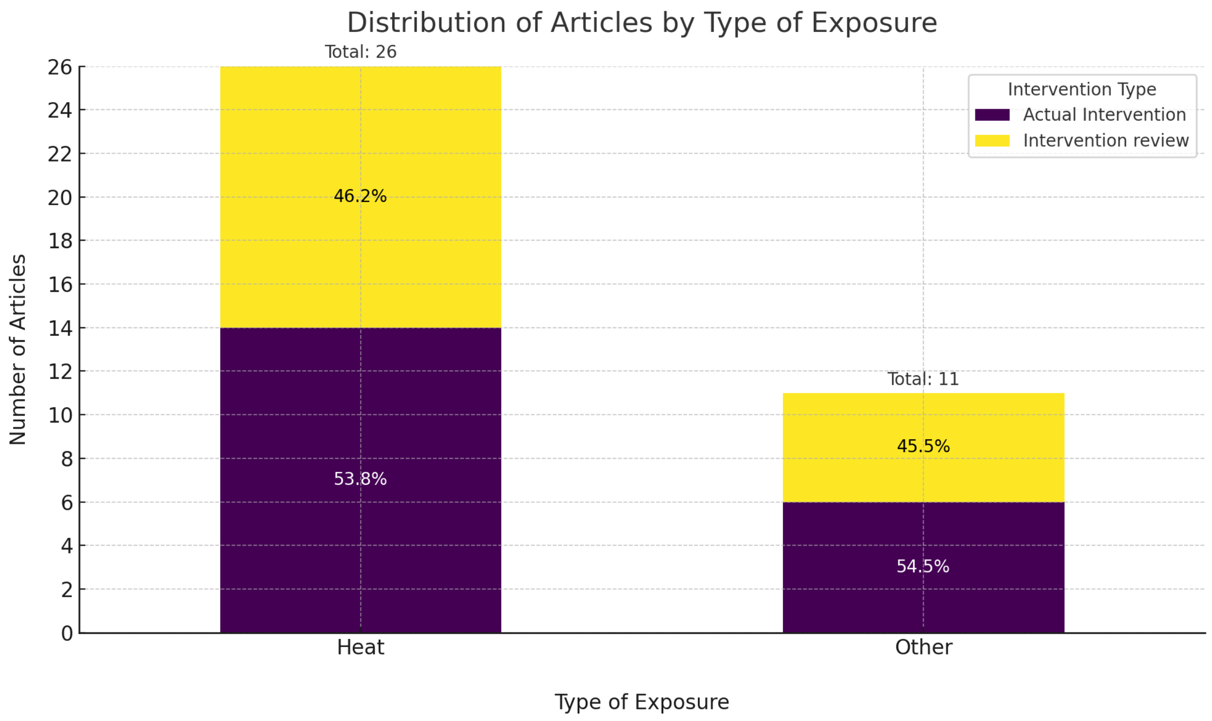
Distribution of Articles by Type of Exposure
Intervention Type
Actual Intervention
Intervention review
Number of Articles
Type of Exposure
Heat
Other
Total: 26
Total: 11
46.2%
53.8%
45.5%
54.5%

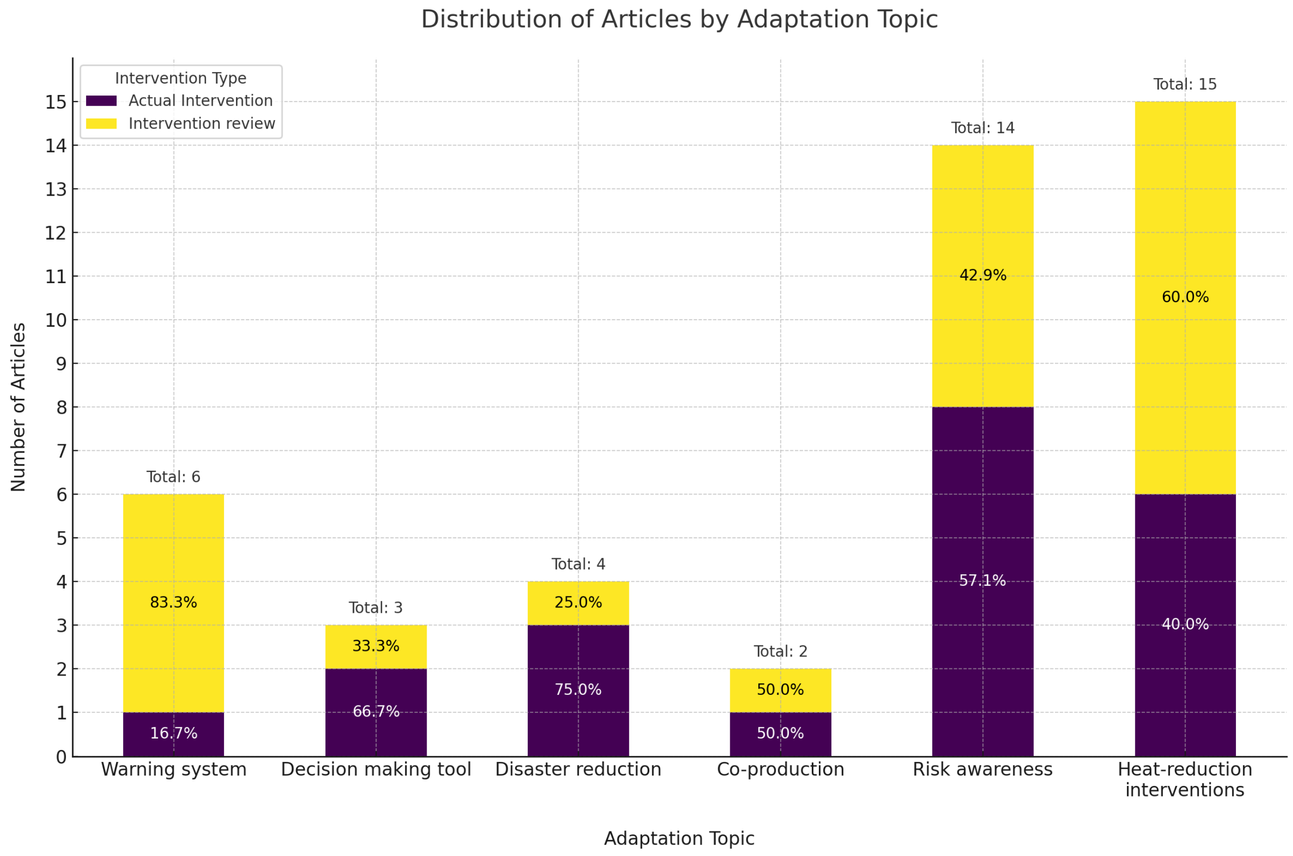
Distribution of Articles by Adaptation Topic
Intervention Type
Actual Intervention
Intervention review
Number of Articles
Adaptation Topic
Warning system
Decision making tool
Disaster reduction
Co-production
Risk awareness
Heat-reduction interventions
Total: 6
Total: 3
Total: 4
Total: 2
Total: 14
Total: 15
83.3%
16.7%
33.3%
66.7%
25.0%
75.0%
50.0%
50.0%
42.9%
57.1%
60.0%
40.0%
