# Supplementary material for: Scoping Review of Climate Change Adaptation Interventions for Health: Implications for Policy and Practice
Source: Int J Environ Res Public Health. 2024 Nov 26;21(12):1565. doi: 10.3390/ijerph21121565 (PMC11675531; doi:10.3390/ijerph21121565)
Supplement: Supplementary file 1 [file ijerph-21-01565-s001.zip › Table S1.pdf]

TABLE S1: DATA EXTRACTION TOOL

**Project name**	
**Article reviewer**	
**Article name**	
**Eligibility**	**Include (actual intervention), include (review of interventions), exclude (not an adaptation study), exclude (not climate change), or query**
**Full citation**	
**Article web link**	
**Research problem addressed by article or main topic covered**	
**Study objective**	
**Geographic region of study**	
**Population group**	
**Temporal focus [date of study, or season if relevant]**	

**Describe the intervention(s)**	
**Methods used to evaluate the outcome**	
**Main results, including any negative or unintended findings**	
**Conclusions**	
**Inequities reported**	
